# Investigating the ageing-Parkinson’s disease nexus: standardisation of in vitro models and techniques by the PD-AGE network

**DOI:** 10.1038/s41531-025-01137-2

**Published:** 2025-10-09

**Authors:** Alexander G. Bury, Alicja Olejnik, Chiara Tocco, Nathalie Saurat, Elezabeth Stephen, Dirk Hockemeyer, Jens C. Schwamborn, Lorenz Studer, Pier Giorgio Mastroberardino, Silvia Bolognin, Tilo Kunath, Viktor I. Korolchuk, Janelle Drouin-Ouellet, Heather Mortiboys

**Affiliations:** 1 Sheffield Institute for Translational Neuroscience (SITraN), School of Medicine and Population Health, University of Sheffield, Sheffield, UK; 2 Faculty of Pharmacy, University of Montreal, Montreal, QC Canada; 3 The Center for Stem Cell Biology, Sloan Kettering Institute for Cancer Research, New York, NY USA; 4 Developmental Biology Program, Sloan Kettering Institute for Cancer Research, New York, NY USA; 5 Australian Regenerative Medicine Institute, Monash University, Clayton, Australia; 6 Department of Molecular and Cell Biology, University of California, Berkeley, Berkeley, CA USA; 7 Innovative Genomics Institute, University of California, Berkeley, Berkeley, CA USA; 8 Chan Zuckerberg Biohub, San Francisco, CA USA; 9 Developmental and Cellular Biology, Luxembourg Centre for Systems Biomedicine, University of Luxembourg, Belvaux, Luxembourg; 10 Department of Molecular Genetics, Erasmus MC Cancer Institute, Erasmus University Medical Center, Rotterdam, Netherlands; 11 Department of Life, Health and Environmental Sciences, Università degli Studi dell’Aquila, L’Aquila, Italy; 12 MERLN Institute for Technology-Inspired Regenerative Medicine, Maastricht University, Maastricht, The Netherlands; 13 Institute for Stem Cell Research, School of Biological Sciences, The University of Edinburgh, Edinburgh, UK; 14 Biosciences Institute, Faculty of Medical Sciences, Newcastle University, Newcastle, UK

**Keywords:** Biological techniques, Computational biology and bioinformatics, Neuroscience, Stem cells

## Abstract

Ageing is the primary risk factor for Parkinson’s disease, yet the intricate interplay between these processes remains ambiguous. This position paper, a collaborative output from the PD-AGE consortium, addresses the urgent need for standardising methods in in vitro modelling. A panel of international experts recommends human induced pluripotent stem cell (iPSC)-derived models, with chemically induced ageing methods, such as the SLO cocktail, as a robust system. Furthermore, the consortium highlights the value of direct and semi-direct reprogramming for retaining donor-specific ageing phenotypes. The paper also outlines a prioritised panel of measurable parameters, categorised into senescence, inflammaging, omics profiling, and mitochondrial dysfunction, providing a consistent framework to enhance research reproducibility, investigating the nexus of ageing and Parkinson’s. In addition, we provide links to SOPs (10.5281/zenodo.15056603) [1] to measure the key measurable ageing parameters outlined in this review to facilitate consistency and reproducibility within the field.

## Introduction

### Parkinson’s disease epidemiology

Parkinson’s disease (PD) affects 0.3% of the global population, 1% of the over-60s and 5% of the over-80s population – this reflects the fact that ageing is the chief risk factor of PD^1,2^. PD is best characterised by its motor symptoms: a resting tremor, postural instability, rigidity and bradykinesia; resulting from the neurodegeneration of dopaminergic (DA) neurons in the *Substantia Nigra pars compacta* (SNpc)^3^. Non-motor symptoms, which are less well characterised, include: impaired REM sleep, cognitive dysfunction, depression and anxiety are more associated with neurodegeneration of non-dopaminergic neuronal populations^4,5^. A main pathological hallmark of PD is the aggregation of misfolded α-synuclein (aSyn), which is incorporated within Lewy Body structures. The exact role of Lewy bodies in contributing to, or otherwise ameliorating, PD pathogenesis caused by toxic soluble a-Synspecies, remains unresolved^6^. DA neurons are the most affected neuronal population in PD, but progressive loss of this subtype in the SNpc is also shown to occur in normal ageing^7–12^. This age-associated decline in DA neurons has been shown by a reduction in tyrosine hydroxylase (TH) staining in the SNpc of healthy, aged non-human primates^1,13^. This also suggests that though there are shared mechanisms of neurodegeneration, particularly of DA neurons in PD and ageing, they appear to be more pronounced in PD^6,14^,

### Parkinson’s disease and ageing

There are multiple shared mechanisms in ageing and the pathogenesis of PD, including dysregulated autophagy, genomic instability, telomere attrition, impaired proteostasis, senescence, epigenetic modulation, inflammation, impaired intercellular communication, nutrient sensing, microbiota and mitochondrial dysfunction^15,16^. Of these mechanisms, mitochondrial dysfunction, dysregulated proteostasis, inflammation and cellular senescence show the greatest degree of overlap between PD and ageing^14^. However, the molecular intricacies underlying these two distinct processes remain unclear.

### Mitochondrial dysfunction

Mitochondrial dysfunction in PD is linked to reduced electron transport chain complex I activity, which has been observed in PD patient nigral tissue homogenate^17^ and PD patient-derived fibroblasts^18,19^. Mitochondrial dysfunction is also implicated in PD through an increase of somatic mitochondrial DNA (mtDNA) deletions^20^ and point mutations^21^, in the SNpc of PD patients, resulting in impaired oxidative phosphorylation. MtDNA deletions are also characteristic of pathological ageing within highly metabolic cells, such as SNpc DA neurons^22^. Reactive oxygen species (ROS) are implicated in mtDNA deletion formation. DA neurons are at a higher risk to develop oxidative stress-associated damage, because ROS are generated through oxidative phosphorylation and through DA metabolism^23^. In addition to mutation load, mtDNA damage is independently complicit in PD pathophysiology. Leveraged against a multi-copy genome, mtDNA repair is more rudimentary compared with nuclear (nDNA) and lacks some mechanisms associated with oxidative lesion repair, such as Nucleotide Excision Repair (NER)^24^. Recent imaging and qPCR-based methodologies, measuring mtDNA lesion frequency, highlight elevated oxidative mtDNA damage in human post-mortem tissue^25,26^ Peripheral Blood Mononuclear Cells (PBMCs)^27^ and a human induced Pluripotent Stem Cell (hiPSC)-derived neuronal model of familial PD^25^. The exact role of mtDNA lesions in PD pathophysiology is not fully understood, but it has been proposed that they could impact mitochondrial homoeostasis, as evidenced by the increase in mtDNA biogenesis as a compensatory mechanism primarily in SNpc DA neurons^28^ or trigger an inflammatory response^29–31^.

### Inflammaging

The combination of mitochondrial dysfunction, elevated ROS, and proteotoxicity, associated with overloaded protein degradation systems, are drivers of inflammation in PD and ageing^32^. Chronic inflammation, termed “inflammaging”, is another hallmark of ageing and neurodegeneration triggered by damage-associated molecular patterns (DAMPs), such as ROS, ATP and extracellular mtDNA. This results in the production of cytokines and further oxidative species, which directly damage cells and tissues^33^. Inflammatory markers, such as IL-6 and IL-8, also contribute to the senescence-associated secretory phenotype (SASP), which leads to the induction of cellular senescence^34,35^. Cells with a higher metabolic threshold, such as DA neurons, are most susceptible to chronic inflammation and are particularly prone to induced senescence^36^. PD is heterogeneous and arises from a number of genetic and environmental factors^26,37^. A recent study suggests that inflammaging may be specific to industrialised populations^38^, whilst other work suggests that inflammatory responses with age are common within species^39,40^. This supports and suggests both ageing and PD are heterogeneous and influenced by environmental factors in a manner that is human specific^41^.

### Senescence

Senescence in mitotic cells is associated with irreversible cell cycle arrest in response to oncogenic stressors such as DNA damage^42,43^, telomere shortening^44^ and epigenetic perturbations^45^. Senescence is initiated by p16 or p21 cyclin-dependent kinase inhibitors, which trigger cell cycle arrest in response to the DNA damage response or telomere attrition^46,47^. Though the mechanistic basis of senescence is less well defined in neurons, relative to mitotic cells, a senescent phenotype has been reported in mouse primary Purkinje neurons through p21 in response to DNA damage and pro-inflammatory factors^48,49^. This p21-dependent senescence phenotype has, to our knowledge, not been investigated in PD, although a study has reported an increase of p21+ cells in the midbrain of PD patients, and that loss of SATB1, a DNA-binding protein, could induce p21-dependant cellular senescence in iPSC-derived DA neurons^36^. Whilst p16 levels have been shown to be elevated in PD^32^ and some studies have suggested telomere attrition is predictive of PD progression and severity^50,51^, contradictory data and lack of consensus on the role of telomere length in PD aetiology, limits the use of telomere length as a robust biomarker of PD in the context of ageing^52–54^. SASP comprises a number of factors that contribute to the senescent phenotype at the cellular level, these include growth factors, chemokines and cytokines, the latter of which can also act in paracrine fashion, spreading senescence to neighbouring cells^34^. The senescent phenotype is also characterised by mitochondrial-dependent ROS generation^55^, the accumulation of senescence-associated beta-galactosidase (SA-β-gal) in the lysosomes^56,57^, senescence-associated heterochromatic foci (SAHF)^58^ and phosphorylation of the histone protein H2AX (γH2AX) in response to double-stranded DNA breaks^59^. Biomarkers associated with senescence such as SA-β-gal activation, SASP induction, loss of lamin B1, γH2AX foci and oxidative stress have been observed in aged^36,49,55,60–65^and paraquat-induced Parkinsonian mouse models^32^. SA-β-gal and SASP biomarkers have also been observed in rat^66–69^ and non-human primate models of ageing^70,71^.

### Disparities between Parkinson’s disease and ageing

Whilst there is clear evidence supporting the association between PD and ageing, there are notable differences in reported changes in DA neurons in Parkinsonian and aged individuals. These include: the number of neurons, levels of oxidative species, αSyn pathology, microglial activation, proteasomal and lysosomal dysfunction. This suggests that the interaction between ageing and PD pathophysiology is complex and not fully understood^1,72^. To better understand common and distinct mechanisms between ageing and PD, it is necessary to standardise the way in which we conduct research in both the fields of ageing and neurodegeneration. This includes recognising the most appropriate disease model(s) and selecting an appropriate panel of biomarkers to best investigate common pathways.

### Cellular models of PD and ageing

Whilst animal models are an important tool to understand the mechanistic basis of ageing and PD, a key limitation of animal models is that PD is a uniquely human disease. The time taken for features of PD to manifest necessitates the use of exogenous induction of certain aspects of PD pathophysiology in animal models^73–77^ and no animal can adequately model all facets of PD simultaneously^41^. Cells sampled from patient peripheral tissues, such as PBMCs, allow more discrete means to assay human tissue biomarkers, but data have so far had limited reproducibility^78,79^. Fibroblasts can also be cultured from patients and age-matched donors and retain age-associated characteristics, although many features of the PD pathophysiology are less pronounced or not expressed in fibroblasts compared to neurons^18,80–82^. Features of PD pathology that have been successfully modelled in fibroblasts include mitochondrial dysfunction and turnover^19,83–88^, lysosomal dysfunction^18,89–93^ and inflammation^14,35,94^.

The discovery and use of “Yamanaka” transcription factors to convert human fibroblasts into hiPSCs, which can then be differentiated into neurons, provides a means to model PD and age-associated disease in a human-based system^95–98^. Since then, a number of cell reprogramming strategies have become available for the conversion of human fibroblasts into neural cells: the differentiation of reprogrammed hiPSCs, the differentiation of reprogrammed induced neuronal progenitor cells (iNPCs)^99,100^ and finally direct reprogramming from fibroblasts into neurons^101–103^ and astrocytes^104^.

Small molecules can be used to differentiate hiPSCs into DA neurons, which express pan-neuronal markers such as βIII-tubulin and the DA machinery, including the marker TH^105,106^. Subsequently, a range of hiPSC-derived neuronal models of idiopathic and familial PD have been derived from patients, including from patients harbouring *SNCA*, *PINK1*, *PRKN*, *LRRK2* and *GBA* mutations^80,96,107–120^, as well as sporadic cases^97,114^. Transcription factors, such as Neurogenin 2 (NGN2), can also be used to generate a high induced neuron yield rapidly, bypassing the neuronal progenitor stage^121,122^. Using this approach in combination with other transcription factors or small molecules, the generation of iDAs has been possible^123–125^. A further refinement of this methodology is the use of doxycycline-induced NGN2, which improves efficiency and reduces batch heterogeneity^126^. A next step after the development of neuronal models was brain organoids, where midbrain organoids are of particular relevance for PD^127–132^. More recently, the level of complexity of 3D models have improved, with assembling of midbrain and striatal organoids to mimic the nigrostriatal pathway, as well as with the experimental induction of cellular ageing^133^. Like iPSC-derived neuronal differentiation, which provides the basis for differentiated organoid models^131,134^ cocktails of growth factors and small molecules can be used to differentiate stem cells into cells of a specific tissue type – such as midbrain neuronal populations^135–137^. Organoid systems offer the potential to model a number of disease features of PD and pathological ageing – including mitochondrial dysfunction, senescence, neuro-inflammation and omic-signatures^138,139^, Whilst organoids themselves are beyond the remit of this article, they do offer long term potential for modelling ageing and neurodegenerative disease. The standardisation of two-dimensional models will only serve to facilitate the development of organoid models going forward.

A notable consequence of the reprogramming process into pluripotency is the loss of cellular ageing signatures, including age-associated changes in DNA methylation patterns and histone modifications, and telomere shortening, which can affect the suitability of this approach in modelling certain aspects of cellular ageing and neurodegenerative diseases^140,141^. Other key age-associated features lost during rejuvenation are the progressive impairment in oxidative phosphorylation^142^, and the age-associated impairment in autophagy^143^. To overcome this limitation, researchers have developed several strategies to induce features of ageing in iPSC-derived cells. These include: long-term culturing^144^ and induced telomere shortening^145^.

By reprogramming terminally differentiated cells directly into somatic cells of another tissue, it is possible to circumvent the pluripotency stage^101–103^, a methodology termed ‘direct cell reprogramming’. Lineage-determining transcription factors can be used to reprogramme somatic cells into subtype-specific neurons, including DA neurons^111,143,146^.

The use of Yamanaka factors supplemented with neural transcription factors, can be used to reprogramme somatic cells into tri-potent induced neural progenitor cells (iNPCs) – which can be differentiated into neurons, astrocytes or oligodendrocytes^99,100^. This process of ‘semi-direct reprogramming’ differs from ‘direct reprogramming’ because somatic cells are first differentiated into progenitor cells, bypassing pluripotency, before being terminally differentiated into cells of a different lineage. This introduces an intermediate step in the differentiation process with the possibility of cryopreserving progenitor cells. The differentiation of human fibroblast-derived iNPCs into iDAs has been demonstrated for the investigation of metabolic and mitochondrial dysfunction in Parkinson’s disease^18,147,148^. Importantly, dermal fibroblasts harbour an endogenous heterogeneity^149^, which can lead to issues related to the clonal nature of iPSCs, which is not the case when using direct and semi-direct reprogramming. However, inherent inter-individual variability can impact the yield and reprogramming efficiency of directly reprogrammed cells^143,150,151^, or result in phenotypically immature neurons^152^. Overall, directly or semi-directly reprogrammed cells are less characterised than iPSC-derived cells. For all reprogramming methods, the somatic mosaicism of the starting cell type can affect the resulting cells. Dermal fibroblasts are thought to have more mosaicism than PBMC’s for example; however, a comparison of the starting cell type for reprogramming is beyond the scope of this review. Furthermore, the composition of the culture can change over time, especially with extended periods of culture. Hence, it is important to monitor the cellular makeup of the cultures on which the experimental assays have been performed on.

### The PD-Age network

Accurately measuring ageing in the cellular models of PD is a complex challenge, which demands a collaborative, interdisciplinary approach. The PD-Age network fosters partnership and knowledge sharing among researchers to identify the most valid, reliable and scalable methodologies of assessment of the interplay between PD and ageing. Working group 2 emphasised the pressing necessity for robust and standardised methods to elucidate the overlapping mechanisms to establish the best practices for incorporating ageing into patient-derived cellular models used for PD research. With the aim of harmonising methodologies across studies, the group discussion was divided into two main objectives.

#### The PD-age network: standard operating procedures development

Firstly, the PD-Age Network developed rigorous methodological frameworks of the Standard Operating Procedures (SOPs) utilised for assessing key cellular processes involved in ageing and PD^153^. The group reached a consensus on the importance of methodological precision of SOPs for measuring senescence, inflammaging, telomere length detection and mitochondrial function. The group discussed which pathways should be included in the SOP list, deciding to focus on pathways that had wide applicability and would be accessible to a wide number of labs worldwide. Detailed protocols for assessment of these changes were developed cooperatively, including equipment and reagents necessary, software and step-by-step experimental procedures. This selection of cellular mechanism categories is not exhaustive but does reflect a representative cross-section of pathways that are compatible with commonly available methodologies and align with the collective expertise of the working group members. Moreover, various factors were taken into consideration when deciding on the relevant methods, including but not limited to: feasibility and scalability, sensitivity, reliability, practicability, ease of adoption and robustness across sites and levels of expertise required. These SOPs can be found at: 10.5281/zenodo.15056603^153^.

#### The PD-age network: choosing the reprogramming route

The second focus of this working group was to develop structured and consistent experimental frameworks for measuring PD- and age-relevant changes in vitro to minimise challenges which may be associated with it. This was done via workshops, questionnaires and literature reviews, to reach consensus on the most appropriate cellular reprogramming models and biomarkers for investigating both ageing and PD. Several measurable markers have been observed in cellular models of PD and ageing, which can largely be categorised as either inflammatory^154^, metabolic^155^, (multi)omic^156^ or senescent^157^. This part of the discussion centred on the importance of selecting the appropriate method to ensure that the cellular model captures the age-dependent vulnerabilities, which characterise PD pathology, while also recapitulating the disease phenotype. The working group undertook an in-depth comparison of iPSC (and iPSC with exogenously induced ageing phenotype), direct and semi-direct routes of reprogramming, to ensure the most appropriate method is chosen.

Here, we document the outputs from these sessions, made up of a panel of researchers with expertise in ageing and PD, to facilitate the standardisation of methodologies for the benefit of researchers of all experience levels wishing to conduct research into the impact of ageing on PD progression.

### Choosing cell reprogramming route

#### iPSCs as a versatile tool for modelling neurodegenerative diseases

To date, many protocols have been established to differentiate iPSCs into various cell types of the brain, utilising developmental signalling cues—such as proteins, small molecules, and transcription factors—that are active during embryonic development. Although efficiency and best practices to differentiate various lineages of neuronal and glial cells have not been discussed by the panel, we recommend the book “Induced Pluripotent Stem Cells - Methods and Protocols”, for material and reagents, step-by-step protocols, and troubleshooting strategies^158^. Since differentiation protocols mimic natural developmental trajectories, cells differentiated from iPSCs typically resemble primary cell types more closely than those that are directly reprogrammed from fibroblasts.

Due to their pluripotent nature, iPSCs can be expanded in vitro prior to differentiation, thus resulting in a high yield of both pluripotent and differentiated cells, and the possibility to scale-up experiments to perform large-scale screens, deep phenotyping experiments or large-scale omics-related studies. As a direct consequence, iPSCs have been characterised in detail, and in some instances, their differentiation paths have been better described than the corresponding processes that control direct reprogramming. Therefore, iPSCs constitute the method of choice when setting up co-culture experiments^159,160^, microfluidic-based organ-on-a-chip cultures^161–167^ and 3D cultures^168–172^. The advent of efficient gene editing methods expanded iPSC’s versatility, allowing the generation of genetically modified cell lines carrying known pathological mutations or risk variants or correcting such mutations in patient-derived iPSCs, to produce isogenic control lines^173^. Finally, iPSC-derived precursor cells show great structural and functional integration when engrafted, and have been used in clinical trials, further consolidating their biological and translational relevance^57,174–176^.

#### Induction of cellular ageing in iPSC-derived cells: where do we stand

Historically, the most used methods were based on replicative stress^17^, ionizing gamma-ray irradiation^177^ or ectopic expression of progerin^95^, a truncated, pathogenic version of the nuclear lamina protein Lamin A. Although very efficient, those methods present caveats and limitations that weaken their relevance in the study of brain ageing in neurodegenerative diseases. For instance, replicative stress is not compatible with post-mitotic cells such as neurons, which also exhibit a high resistance to ionizing gamma-ray irradiation. While the progerin-based approach affects the nuclear envelope, it does not reproduce epigenetic reprogramming, and it has been shown to lack the full complexity of age-related epigenetic drift^178^. Moreover, this approach can trigger acute cellular stress, such as apoptosis and rapid senescence, which may mask or exaggerate the induced ageing and disease phenotypes.

**Fig. 1:**
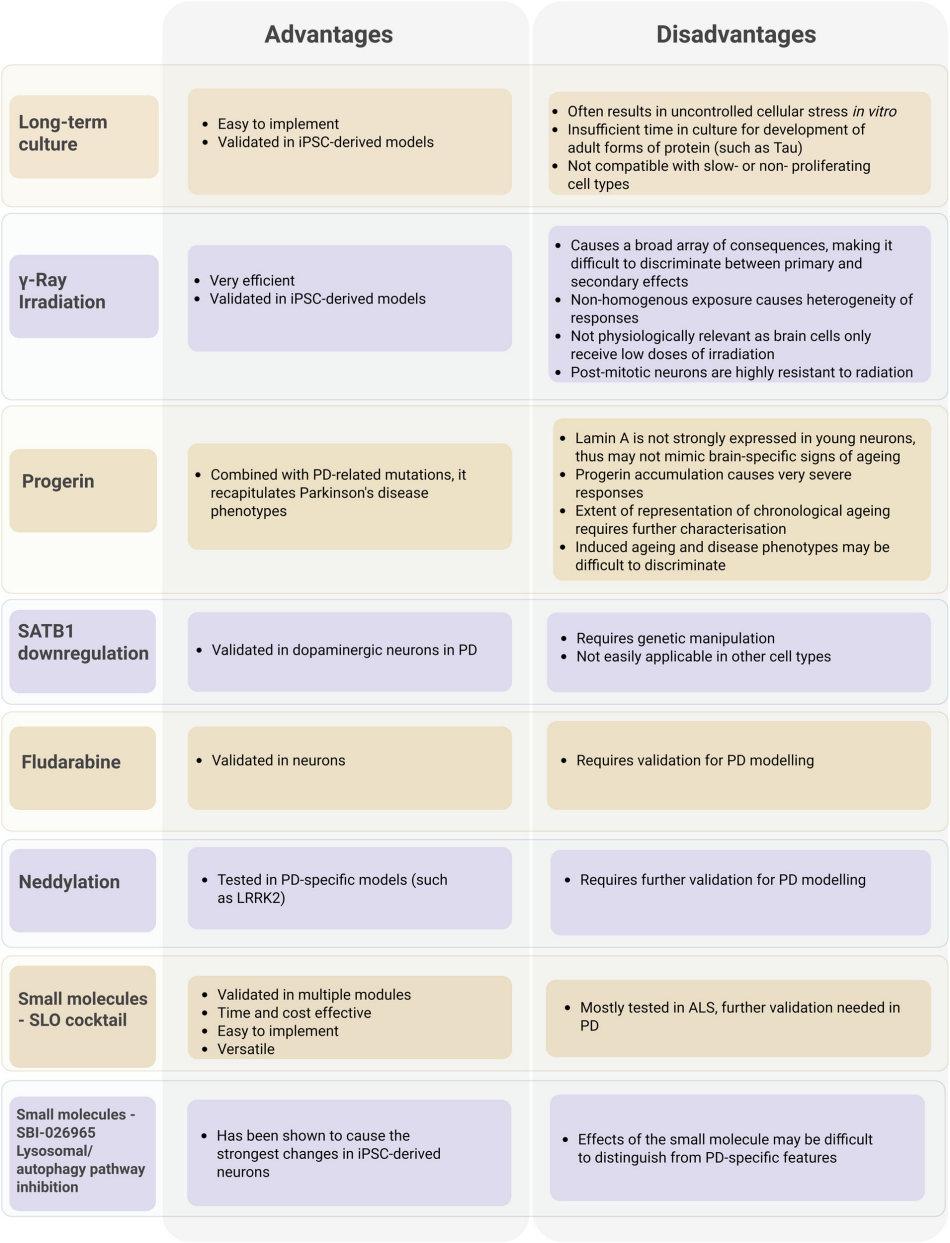
Methods of age induction in iPSC-derived models of PD. Each method is defined as follows: Long-term culture^17^, cells are maintained in culture and frequently passaged until their proliferative potential is exhausted; γ-Ray irradiation^177^, sublethal doses are used to induce DNA-damage and trigger senescence; Progerin^95^, the ectopic expression of this mutant form of lamin A is used to mimic the effect on cells of the Hutchinson-Gilford Progeria Syndrome; SATB1^179^, genetic downregulation of this chromatin remodeller in the context of PD leads to the activation of key senescence genes and ageing pathways; Fludarabine^180^, can cause signs of ageing in hiPSCs by interfering with DNA synthesis; Neddylation^181^ loss of function in iPSC-derived neurons leads to increased hallmarks of ageing and exacerbates neuronal loss in AD and PD neurons; Small molecules^182,185^ that pharmacologically target autophagy, exclusively (SBI-026965) or in combination with nuclear lamina formation and DNA repair (SLO cocktail), induce signs of ageing in iPSC derived neuronal and glial cells.

More recent studies have identified alternative methods to induce ageing in iPSC-derived models. It has been proposed that genetic inactivation of SATB1^179^, a transcriptional regulator whose expression is reduced in DA neurons of PD patients, could be used to investigate the drivers of ageing specifically occurring in PD as opposed to the broad ageing phenotype. RNage^180^, an RNA-seq-based method to calculate ageing scores, can be used to both validate and compare existing protocols and as a screening tool to identify novel strategies. When used to study gene expression profiles from cells treated with several hundreds of compounds, it showed that a few of them, including Fludarabine, could induce an increased RNage score, and cause typical markers of cellular ageing. Similarly, a CRISPR-based whole genome screening^181^ can also be used to identify regulators of ageing. This approach in iPSC-derived neurons helped identify the neddylation pathway as a potential regulation of ageing in Alzheimer’s disease (AD) and could be used to model late-onset phenotypes in PD models. Although promising, these techniques need to be validated in different systems, and further optimization is also required. All methods, along with a more detailed description of their specific pros and cons can be found in Fig. 1.

Among the discussed strategies, we identified the administration of small molecules targeting known ageing-related molecular pathways as the most relevant method to induce ageing in iPSC-derived models of PD^182^. This strategy has multiple advantages: it is easy to use and accessible, time- and cost-effective and very versatile, as the used compounds and their dosage can be adapted based on their relevance to the cell type of choice and the disease to be modelled. Furthermore, simultaneously targeting multiple pathways better mimics the effects of ageing on overall cell health, thus providing many features associated with ageing. To date, the most promising treatment is the SLO cocktail, which combines three molecules, SBI-0206965, Lopinavir and O-151, that respectively target autophagy, Lamin A biogenesis and DNA glycosylase and together base excision repair^183,184^. Defective autophagosomes lead to impaired mitochondrial clearance and increased oxidative stress, whereas DNA glycosylase and Lamin A biosynthesis impairment affect nuclear architecture and lead to DNA damage accumulation. Although it has not yet been tested on DA neurons, the SLO cocktail has been successfully applied to age iPSC-derived cortical neurons^182^, and human microglia^185^, suggesting the method has the potential to be used with many other PD-relevant cell types. As such, the working group recommends the SLO cocktail treatment as the preferred method to age iPSC-derived brain cells.

### Preserving the ageing signature with semi-direct and direct reprogramming

Although our working group established that iPSC-derived models with accelerated ageing should be the system of choice to study the effects of ageing on PD, there are a few instances where preserving the ageing signature of the donor should be preferred. Neurons and astrocytes directly or semi-directly reprogrammed from patient-derived skin fibroblasts maintain the ageing signature of the donor^143,186,187^.

Directly reprogrammed induced neurons (iNs) retain their age-associated epigenetic and transcriptomic signatures, Oxidative Phosphorylation (OXPHOS) and autophagy impairment^143,187–190^, DNA damage^143,187^ and expression of mature TAU isoforms^143,191^. A select number of studies using directly or semi-directly reprogrammed iNs have successfully modelled mitochondrial and lysosomal dysfunction associated with ageing or neurodegenerative disease, including PD^18,142,143,148,187,188,192,193^.

**Fig. 2:**
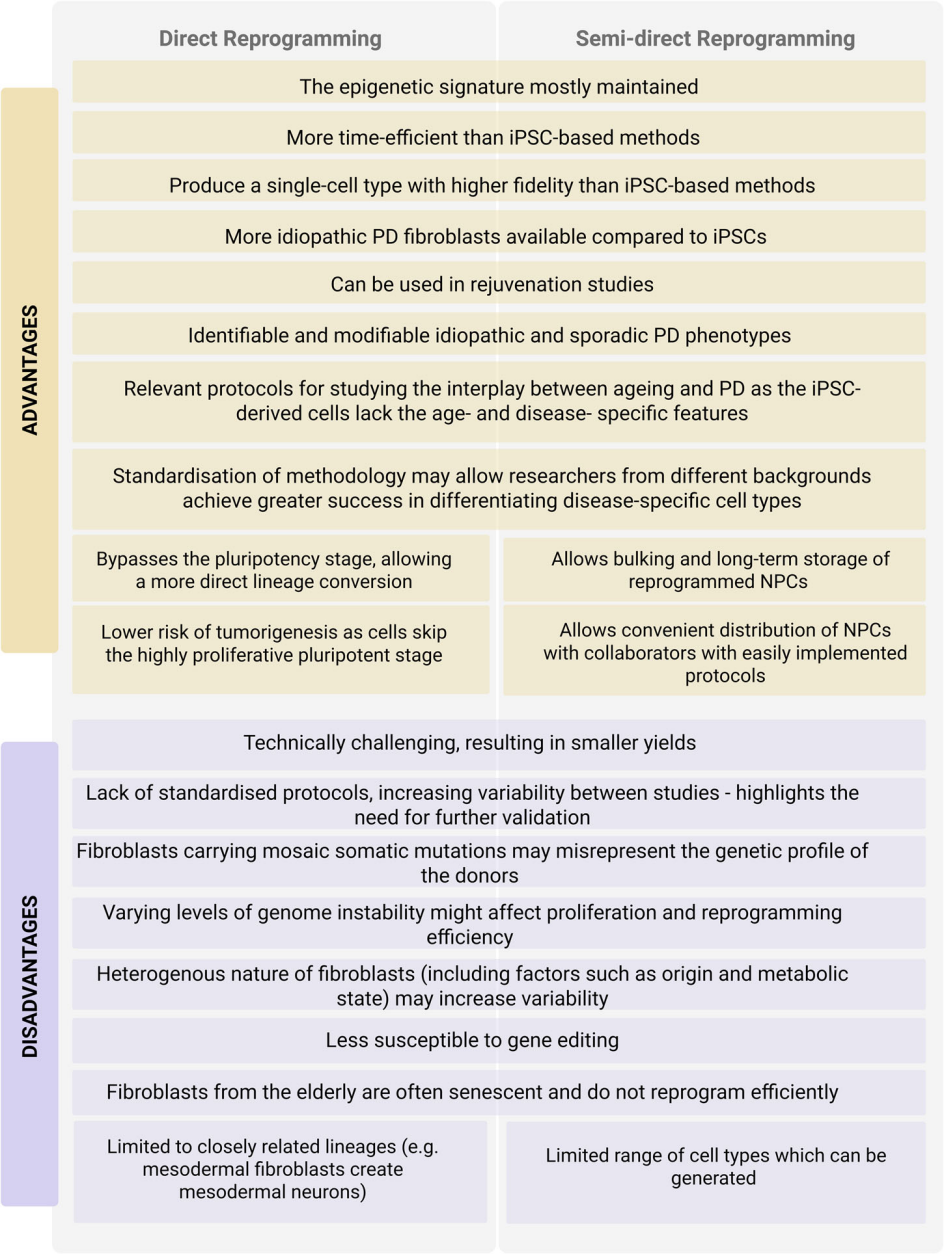
Advantages and disadvantages of direct and semi-direct reprogramming in the study of ageing in PD.

By replicating the exact ageing profile in a patient-specific matter, these strategies represent a powerful tool to study disease mechanisms in an ageing context. However, direct reprogramming methods were not selected as the preferred reprogramming route because of the current challenges associated with their use. Since iNs become post-mitotic early in the conversion process^194^, any study necessitating a high neuronal yield requires extensive expansion of fibroblasts, which can lead to replicative senescence or metabolic changes in parental cells impacting on the reprogramming efficiency and the generated cell product^195^. However, semi-reprogramming methods effectively overcome yield limitations, enabling the production of large quantities of neurons^148^. Heterogeneity between batches^14,143^ and lack of protocol standardisation are also prominent limitations of these models. A detailed list of advantages and disadvantages of direct- and semi-direct reprogramming is reported in Fig. 2.

A feature of PD pathology and ageing not successfully modelled by any of the cell reprogramming methods described so far, is the interaction between distinct cell types within the brain. This could potentially be achieved by the co-culture of different reprogrammed cell types, but success in this area has thus far been limited^104,163,186,196–199^.

In conclusion, iPSC-derived models, and direct and semi-direct reprogramming all present advantages and disadvantages (Fig. 1). While direct and semi-direct reprogramming is generally more time and cost-effective and present the clear advantage of retaining the donor ageing signature, iPSC-based models are overall more standardised, high-throughput and versatile. Thus, selecting the appropriate method should be driven by the specific objectives and needs of the study at hand.

## Assessing ageing in cellular models

### Selection and prioritisation of ageing assays

**Fig. 3:**
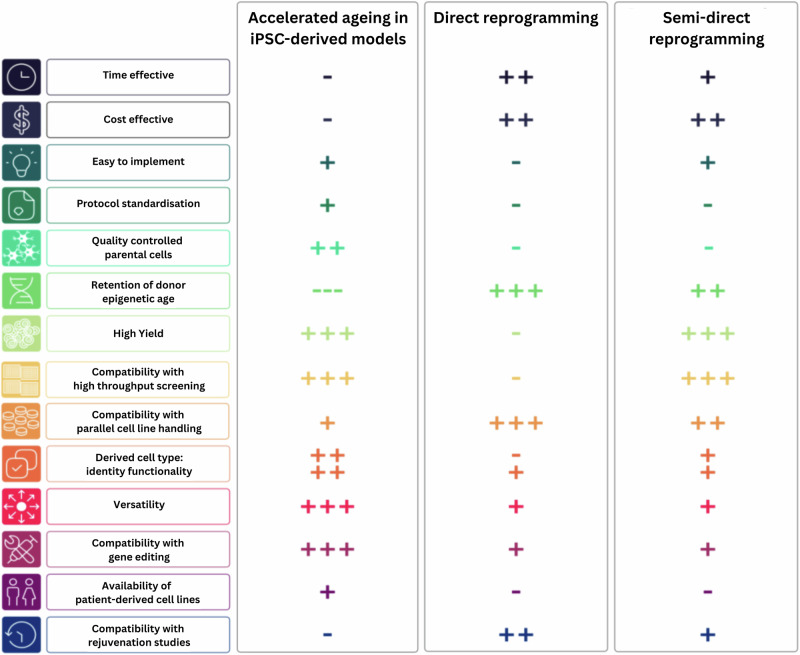
Models of ageing: strengths and weaknesses. Each criteria is defined as follows: time effective, how quickly the method produces the cell type of interest; cost effective, relative expense of the approach; easy to implement, overall complexity of the method; protocol standardization, availability of standard procedures; quality controlled parental cells, ability to maintain high-quality cells without unwanted mutations or inconsistencies, and accessibility of quality control assays; retention of donor epigenetic age, whether the method preserves age related epigenetic modifications; high yield, efficiency of producing a large number of viable cells; compatibility with high throughput screening, assesses if the method can be used for large-scale drug and guide screening and automated testing; compatibility with parallel cell line handling, easiness to process multiple cell lines at the same time; derived cell type identity and functionality, whether the produced cells accurately resemble there in vivo counterpart; versatility, ability to produce a variety of different cell types; compatibility with gene editing, how well the method supports genome editing techniques; availability of patient-derived cell lines, assesses the availability of cell lines derived from human patients, as well as centralised cell banks and depositories; compatibility with rejuvenation studies, whether the method is suited to test strategies to reverse cellular ageing. The + represents if the reprogramming method has this criterion, with more + the better. – represents the method does not have that criterion.

To comprehensively evaluate assays that can be used to evaluate cellular age in in vitro models of PD, the working group identified commonly used assays across four key areas: senescence and inflammaging, omics profiling and mitochondrial function. These key areas were prioritised because they have been clearly linked to ageing^16,200,201^ but, except for mitochondrial function, remain distinct from the aetiology of heritable PD^202^. Subsequently, the collective expertise of the working group members was surveyed to establish a prioritised list of assays that are robust and can be used to validate ageing phenotypes in cellular models. Figure 3 summarises the selected tests and their corresponding functionalities.

To further support researchers looking to perform these assays in their own laboratories, we have generated a standardised web platform to share protocols (SOPs) for key assays. Standardisation will help ensure consistency and reproducibility across research groups and provide guidance when there are multiple different methods that can be used to measure an age-related change The web platform can be found here: 10.5281/zenodo.15056603^153^.

### Senescence and Inflammaging

**Fig. 4:**
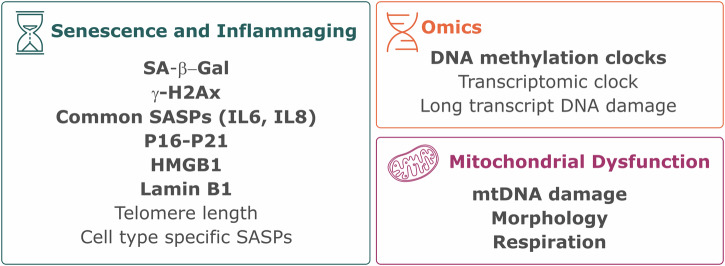
Outline of the key measurable age related cellular changes recommended by the consortium. SOPs for the methods in bold can be found at 10.5281/zenodo.15056603^153^.

Cellular senescence phenotypes are highly heterogeneous and vary based on both cell type and the senescence-initiating stimulus. As such, there is no single assay that can be used to define senescence; rather a combinatorial approach should be used with careful consideration paid to cell type (Fig. 4). Notably, neurons require a tailored approach as they are post-mitotic and therefore it is not appropriate to measure senescence using assays that are directly tied to replication potential. Following discussion by the working group, we recommend prioritising SA-β-Gal (fluorescent probe), γH2AX (immunocytochemistry) and SASP (ELISA) when establishing assays to measure senescence and DNA damage. The group recognised this is not an exhaustive list and additional assays that can be used to further strengthen evidence of senescence are outlined in Fig. 4 and include p16 and p21and loss of HMGB1 and Lamin B1.

Inflammaging is defined as an increase in proinflammatory cytokines as individuals age. Proinflammatory cytokines are also components of SASP. As outlined in Fig. 4, key SASP factors have not been well described across neuronal and glial types and further characterisation and validation are necessary. While IL-6, IL-8, and IL-1β are likely to be relevant, gene expression studies and multiplex cytokine arrays should be used to establish cell type and stimuli-specific profiles of cell types of the brain.

### Omics

Omics-related technologies represent fast-moving and evolving tools to measure ageing. DNA methylation clocks are the most established ageing clocks and the recently published Universal ageing clock^203^ represents an important step forward in using this technology to measure age in cultured cells such as neurons, and glia. We would point researchers wanting to use this tool to the consortia website (https://clockfoundation.org) for further information. Rapid progress is also being made to develop transcriptomic, proteomic and metabolomic-based clocks. Researchers should stay updated with evolving technologies and remain open to the limitations and context-specific applications of these omics approaches.

### Mitochondria in ageing

Mitochondria have critical roles in both ageing and PD. To measure mitochondrial function, it is recommended that the following assays be prioritised by researchers: mtDNA damage Detection, Mitochondrial Morphology Analysis, and Mitochondrial Respiration. However, mitochondrial dysfunction is a key pathology in PD and all the suggested assays have also been used to study PD in the absence of age. Therefore, results should be interpreted carefully, and controls included that allow the impact of the disease model and age to be distinguished.

### Key findings and future directions

Age is the single most important risk factor for PD, but the complexity of the interplay between ageing and PD is yet to be fully determined. The various available in vitro models for this investigation provide a distinct set of advantages and disadvantages. By discussing these properties, the PD-Age network identified an urgent need for methodological rigour to strengthen the understanding of common mechanisms behind ageing and PD.

Out of the wide range of overlapping mechanisms implicated in both ageing and PD studies, this consortium has prioritised protocols utilised for the investigation of senescence, inflammaging, omics profiling and mitochondrial dysfunction (10.5281/zenodo.15056603^153^). Therefore, the standardisation of the in vitro techniques utilised to investigate the underlying pathways will not only reinforce individual studies within this field but also provide a robust framework to minimise variability and improve reproducibility.

While the current most preferred approach to studying PD-related changes, the iPSC cell reprogramming route, is well characterised, the benefits of other in vitro models conserving the epigenetic signature of the donor should be considered. This versatile tool is a method choice of a majority of the in vitro studies into neurodegeneration. With multiple techniques of induction of the ageing features into the iPSC-derived cells, further standardisation is necessary to increase the ability to compare findings across studies. On the contrary, the less characterised practice of obtaining cultured cells carrying the original ageing profile of the donor, have been shown to be a powerful tool for investigating age-related diseases. Despite the advantages of utilising the direct and semi-direct reprogramming approaches of generating cells and maintaining the biological background of the individual biopsy donor, robustness is necessary in the developing procedures. Uniform and efficient protocols will facilitate greater consistency and more accurate comparisons across studies from various institutions.

However, there are multiple outstanding questions future research should continue to explore to advance the ageing research in PD. One of the crucial challenges facing this field is distinguishing the age-specific effects from the PD-specific effects in vitro. This issue, especially vital in distinguishing mitochondrial characteristics and their age- and PD-specific changes will require further elucidation. Secondly, modelling disease progression in the context of ageing still requires refinement, as the current cellular models are unable to fully capture the gradual progression of the disease. Therefore, while the outlined practices may create a solid foundation for ageing and PD studies, avenues such as multi-cellular models, time-lapse investigations and incorporation of risk factors will be critical in the general standardisation across the field.

While the harmonisation of research practices is essential, its challenges should also be considered. For the research community to be able to draw meaningful conclusions achieved from standardised frameworks, the heterogeneity of research settings must be reviewed. Moreover, a key factor to evaluate during this process is the high level of complexity of PD and ageing, their various pathways of pathophysiology and the variability in their presentation across individuals. To overcome this, the process must be adaptable and constantly updated. Also, while great effort was implicated in the selection of methods for measuring the chosen parameters to include the most common laboratory equipment, the differences in technology and resource access may decelerate the standardisation process across regions. Therefore, the PD-Age network emphasises the importance of international partnership and technological unification as indispensable means for establishing these common protocols.

## Data Availability

No datasets were generated or analysed during the current study.
